# Integrated Metabolome and Transcriptome Analysis Reveals the Effect of Anthocyanins on Flower Color Variation in *Michelia odora*

**DOI:** 10.3390/biology15141217

**Published:** 2026-07-22

**Authors:** Yuan Xie, Pengbo Yan, Li Liu, Jun Ni, Shinan Liu

**Affiliations:** 1 Guangxi Colleges and Universities Key Laboratory for Cultivation and Utilization of Subtropical Forest Plantation, School of Forestry, Guangxi University, Nanning 530004, China; 2409392059@st.gxu.edu.cn (Y.X.); 20200099@gxu.edu.cn (J.N.); 2 College of Tourism Ecology and Environment, Guilin Tourism University, Guilin 541006, China; yanpb@gltu.edu.cn; 3 Nanning Arboretum, Guangxi Zhuang Autonomous Region, Nanning 530031, China; liuli.31@163.com

**Keywords:** *Michelia odora*, petal color, anthocyanins, metabolomics, transcriptomics

## Abstract

The flower color of *Michelia odora*, an important landscape tree plant, shows significant ornamental value, but its regulatory mechanisms remain to be further explored. The present work integrated metabolomic with transcriptomic analyses for elucidating the anthocyanin biosynthesis mechanisms in light pink and deep pink *M. odora* flowers. Our metabolomic analysis identified 13 differentially expressed anthocyanins, 11 of which, especially cyanidins, were significantly accumulated within deep pink petals versus light pink ones. As revealed by our transcriptomic analysis, 33 differential genes were related to flavonoid and anthocyanin biosynthesis. Further, transcription factor genes, encompassing three *MoMYB* alongside two *MobHLH* genes, were likely the hub genes that modulated anthocyanin biosynthesis. Quantitative real-time PCR validation indicated that certain genes, like *MoPAL*, *MoC4H*, *Mo4CL*, *MoCHS*, *MoCHI*, *MoUGT75C1*, *MoMYB3*, *MobHLH1*, and *MobHLH3*, displayed concurrent expression with anthocyanin accumulation within deep pink petals. Collectively, these results indicate the role of anthocyanin accumulation in deep pink color, and the effect of upregulated genes on increasing anthocyanin levels. Our results shed novel light on color change mechanisms underlying *M. odora*, and provide a theoretical basis for flower breeding.

## Simple Summary

*Michelia odora* is a native tree species in southern China with high ornamental and economic value. Its flowers usually show a light pink color, but deep pink flowers have also been observed. To understand the reason behind this color difference, we compared the pigments and gene expression profiles between light pink and deep pink flowers. Our results showed that deep pink flowers accumulate more anthocyanins, the pigments responsible for red and pink colors in plants. Several genes involved in anthocyanin biosynthesis were found to be more active in deep pink flowers. These findings indicate that the color variation in *M. odora* is mainly driven by differences in anthocyanin accumulation, regulated at the expression level of genes. This work provides a foundation for further studies on flower color formation in *Michelia* species and offers potential insights for ornamental breeding.

## 1. Introduction

Flower color of plants shows significant biological value, and it is a key trait in evaluating the landscape value of tree plants [1]. Bright colors assist flowers in attracting pollinators including butterflies and honeybees to enhance pollination, increase productivity, and enrich germplasm resources [2]. Flower coloration is an intricate process involving diverse mechanisms, and is potentially the pooled outcome of several factors. Among them, the composition and content of pigments exert important impacts [3]. Pigments that determine flower petal color are anthocyanins, flavonoids, carotenoids, and betalains, with anthocyanins making the greatest contributions [4,5]. Anthocyanins are water-soluble pigments in the flavonoid class of compounds [6], mainly including delphinidin, cyanidin, pelargonidin, peonidin, petunidin, and malvidin. Typically, peonidin is generated via cyanidin methylation, while petunidin and malvidin are produced by delphinidin methylation to varying degrees [7,8,9]. Flower colors, ranging from pink to blue-violet, are tightly associated with various types and levels of anthocyanins. Peonidin and cyanidin display a purple-red color, while pelargonidin shows a brick-red color. Delphinidin, petunidin, and malvidin are between blue and purple [10,11]. Anthocyanins have important physiological effects in plants, such as attracting pollinators, protecting against ultraviolet and pathogen infection, and regulating plant development under biotic/abiotic stresses [12,13,14,15]. Anthocyanins show numerous biological effects on human health and assist in preventing and reducing type 2 diabetes, allergy, obesity, and cardiovascular diseases [16,17]. Due to the multiple activities of anthocyanins, elucidating their biosynthesis mechanisms is of great significance.

Anthocyanin biosynthesis is a conserved process that has been extensively analyzed in plants [18]. According to catalytic performance, the associated genes are classified into early and late biosynthetic genes (EBGs and LBGs, respectively). EBGs, consisting of *PAL*, *4CL*, *C4H*, *CHS*, *CHI*, *F3H*, *F3′H*, and *F3′5′H*, are dominant in the early flavonoid pathway. Phenylalanine is catalyzed by *PAL*, *C4H*, *4CL*, and *CHS* in sequence, forming chalcone. *CHI* is responsible for catalyzing chalcone conversion to naringenin, while *F3H*, *F3′H*, and *F3′5′H* can catalyze the transformation of naringenin to dihydroflavonols (DHK, DHQ, DHM). LBGs including *DFR*, *ANS*, *UFGT*, and *OMT* mainly dominate the later synthesis stages. Dihydroflavonols can be reduced by *DFR* into colorless anthocyanins, which are subsequently catalyzed by *ANS*, producing colored anthocyanins including cyanidin, delphinidin, and pelargonidin. Anthocyanidins are subjected to multiple modifications catalyzed by *UFGT* and *OMT*, finally producing stable anthocyanins [5,12,19]. Furthermore, upregulating or downregulating enzyme-encoding genes dramatically influences the accumulation of flavonoids (like anthocyanins), thereby impacting plant color changes [20,21]. Transcription factors (TFs), like R2R3-MYB, bHLH, WD40, and bZIP, may modulate structural gene levels to enhance or suppress anthocyanin biosynthesis within plants [22,23,24,25]. For example, ectopic *CmMYB11* expression from chrysanthemum within tobacco (*Nicotiana tabacum*) dramatically upregulates transcript levels of anthocyanin biosynthetic genes (*NtCHS*, *NtCHI*, *NtF3H*, *NtF3′H*, *NtDFR*, *NtFLS*, and *NtANS*), and promotes the biosynthesis of flavonols and anthocyanins, giving rise to deep pink petals [26]. *MdMYB16* upregulation within red-fleshed apple callus suppresses *MdUFGT* and *MdANS*, finally suppressing anthocyanin synthesis and changing callus color from red to yellow [27]. The above results suggest the diverse species-specific features of anthocyanin regulatory mechanisms that deserve additional investigations with non-model ornamental tree plants.

The genus *Michelia* in the family *Magnoliaceae* includes many species that have great ornamental value. The molecular basis for flower coloration has been demonstrated in many *Michelia* species. For instance, cyanidin derivatives help generate red pigments in *M. maudiae* [28]. Likewise, cyanidin 3-rutinoside represents the main anthocyanin for forming the purple color of *M. crassipes* [29]. *McMYB52* can modulate anthocyanin biosynthesis through activating *McCCoAOMT* [30]. *4CL*, *PAL*, and *C4H* within *M. alba* can regulate pigment biosynthesis [31]. *MwMYB-1* overexpression from *Magnolia wufengensis* within tobacco and *Arabidopsis* helps accumulate anthocyanins to render red colors of leaves [32]. As demonstrated by the above results, anthocyanin accumulation plays a key role in determining the flower coloration of *Magnoliaceae* species, but the anthocyanin biosynthesis mechanisms may differ across species.

*Michelia odora*, belonging to the *Magnoliaceae* family, is an evergreen species in the genus *Michelia*. It is a valuable native tree in China and is mainly distributed in the southern regions. *M. odora* holds great economic and medicinal significance. Its wood is highly prized for manufacturing premium furniture. Beyond its use in carpentry, its roots, bark, branches, leaves, and fruits can be utilized in the development of pharmaceuticals, fragrances, and cosmetics. Furthermore, as an essential ornamental trait, flower color attracts considerable attention. Vibrant colors not only enhance the status of *M. odora* as a garden species but also increase its economic value in horticulture. The flowers of *M. odora* typically exhibit light pink color, while individuals with deep pink flowers have also been discovered. The specific anthocyanins and genes responsible for color variations in *M. odora* remain poorly understood, which has impeded the development of molecular design breeding technologies aimed at flower color enhancement. Given that anthocyanins function as essential regulators of flower coloration in *Magnoliaceae* species, we hypothesized that differential accumulation of anthocyanins drives flower color divergence in *M. odora*. Consequently, the present work aimed to elucidate the molecular bases governing the expression levels of genes and the production of metabolites associated with flow color in *M. odora* through metabolomic and transcriptomic analyses. The results in this study will lay a theoretical basis for understanding flower color formation in *M. odora* and offer valuable insights for enhancing its ornamental value through targeted breeding. Furthermore, as a case of natural color polymorphism within *Magnoliaceae*, our findings contribute to the broader understanding of anthocyanin-mediated color diversification.

## 2. Materials and Methods

### 2.1. Plant Materials

**Figure 1:**
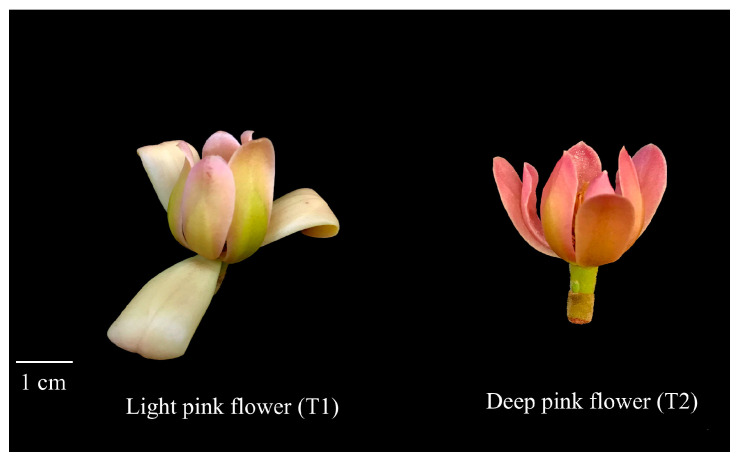
Two colors of *M. odora* flowers: light pink flower (T1) and deep pink flower (T2).

*M. odora* trees bearing light pink or deep pink flowers were cultivated under consistent site conditions at the Nanning Arboretum (108°21′ E, 22°40′ N), Guangxi Province, China. Flowers of two different colors (light pink and deep pink) were collected at the full bloom stage in March 2022 and designated as T1 and T2, respectively (Figure 1). Regarding each flower color, nine flowers (with three flowers in each replicate) were harvested from a single tree for transcriptomic and metabolomic analyses. All fresh floral tissues were immediately flash-frozen in liquid nitrogen after collection and then stored at −80 °C in an ultra-low-temperature freezer for further analysis.

### 2.2. Extraction and LC–MS/MS Analysis of Anthocyanins

According to the methodological description by Hong et al. [33], a tissue grinder (MB-96, Meibi, Pinghu, China) was utilized to grind the freeze samples using zirconium beads for 2 min at 50 Hz. Subsequently, the resulting powder (50 mg) was added into 60% methanol in water (1 mL). After vortexing for 10 min, the extract was subjected to 10 min of centrifugation (12,000 r/min, 4 °C). The aforementioned steps were repeated to re-extract the residue under identical conditions. Thereafter, supernatants were harvested and passed through the 0.22 μm membrane filter (Jinteng) prior to LC–MS/MS analysis.

The Vanquish UHPLC System (Thermo Fisher Scientific, Waltham, MA, USA) was utilized for liquid chromatography (LC) analysis. The ACQUITY UPLC^®^ BEH C18 column (2.1 × 100 mm, 1.7 μm) (Waters, Milford, MA, USA) was employed for chromatography at 50 °C. The flow rate was set at 0.25 mL/min, and the injection volume was 2 µL. For LC–ESI (+) MS analysis, the mobile phases consisted of (A) 1% formic acid in water (*v*/*v*) and (B) 1% formic acid in methanol (*v*/*v*). The following gradient was set for separation: 0.0~2.0 min, 5% B; 2.0~15.0 min, 5~70% B; 15.0~15.1 min, 70~95% B; 15.1~18.0 min, 95% B; 18.0~20.0 min, 95~5% B; 20.0~25.0 min, 5% B.

Metabolites were subjected to mass spectrometry using the Q Exactive mass spectrometer (Thermo Fisher Scientific, USA) with the electrospray ionization (ESI) source. MS1 and MS/MS were simultaneously employed for acquisition in the full mass-ddms2 mode, utilizing data-dependent MS/MS. Our analysis parameters included sheath gas pressure at 47 arb; auxiliary gas flow at 15 arb; capillary temperature at 320 °C; spray voltage at 3.50 kV for ESI (+); MS1 range from *m*/*z* 150 to 1300; MS1 and MS/MS resolving powers at 70,000 and 17,500 FWHM, respectively; number of data-dependent scans per cycle of 4; and normalized collision energies at 10 and 60 eV, with dynamic exclusion time set to automatic [34].

Anthocyanins were identified with high-accuracy mass measurements (<30 ppm), and MS/MS data were matched to several databases, including KNApSAck (https://www.knapsackfamily.com/KNApSAcK/, accessed on 30 December 2022), HDMB (https://hmdb.ca, accessed on 30 December 2022), KEGG (https://www.genome.jp, accessed on 30 December 2022), LipidMaps (https://lipidmaps.org, accessed on 30 December 2022), and PubChem (https://pubchem.ncbi.nlm.nih.gov, accessed on 30 December 2022). The Ropls software version 1.28.0 [35] was utilized for principal component analysis (PCA) and orthogonal partial least-square discriminant analysis (OPLS-DA) for dimensionality reduction of sample data. OPLS-DA enabled the identification of discriminating anthocyanins based on the variable importance in projection (VIP). The student’s *t*-test and the VIP values generated through OPLS-DA and fold change (FC) analysis were employed for identifying contributory variables for classification. Finally, statistical significance of anthocyanins was determined based upon the *p*-value < 0.05 and VIP value > 1 thresholds. The functional annotation of differential anthocyanin metabolites (DAMs) was performed with the KEGG database (https://www.kegg.jp/kegg/compound/, accessed on 30 December 2022), and KEGG pathways were enriched based on the annotation results.

### 2.3. RNA Extraction, Library Construction, and Transcriptome Sequencing

Total RNA isolation was conducted according to the protocol provided by the RNA extraction kit (TIANGEN, Beijing, China). The RNA quality and integrity were evaluated by the NanoDrop (Thermo Fisher Scientific, Waltham, MA, USA) and the 2100 Bioanalyzer (Agilent Technologies, Santa Clara, CA, USA), respectively. Library construction was executed with the NEBNext Ultra II RNA Library Prep Kit for Illumina (New England Biolabs, Ipswich, MA, USA) in line with specific protocols. Double-stranded cDNA was prepared by the reverse transcription kit (Illumina, San Diego, CA, USA) using the purified mRNA fragments as templates. Later, PCR was conducted to amplify the obtained libraries, followed by purification and quality evaluation. Based on effective concentrations, libraries meeting quality control standards were combined, followed by sequencing on the Illumina Novaseq 6000 (Illumina, San Diego, CA, USA) platform with 150 bp paired-end reads. Sequencing data were acquired through capturing and processing fluorescence signals. Firstly, raw sequencing data were processed using fastp (v0.22.0) to remove low-quality reads, adapter-contaminated reads, and reads containing more than 5% ambiguous nucleotides. Second, the clean reads were assembled using Trinity (v2.15.1) to obtain transcript sequences, and the longest transcript for each gene was designated as the unigene.

### 2.4. Gene Functional Annotation and Data Analysis

Gene function was annotated using multiple public databases such as GO (http://geneontology.org/, accessed on 30 December 2022), KEGG (http://www.kegg.jp/, accessed on 30 December 2022), UniProt (http://www.uniprot.org/help/uniprotkb/, accessed on 30 December 2022), and eggNOG (http://eggnog6.embl.de/, accessed on 30 December 2022). The gene expression levels were predicted by RSEM (v1.2.26) [36]. The R package DESeq2 was adopted for identifying differentially expressed genes (DEGs) [37] based upon the |log2FC| ≥ 1 and *p*-value < 0.05 thresholds [38]. GO functional annotation of DEGs was implemented with GOseq (v1.10.0) package, whereas KEGG pathway analysis was conducted with KOBAS (v2.0.12) software.

### 2.5. Analysis of MYB and bHLH TFs Related to Anthocyanin

TFs in *M. odora* were predicted through comparative analysis against the PlantTFDB database (https://plantTFDB.gao-lab.org/, accessed on 30 December 2022). R2R3-MYB and bHLH proteins associated with anthocyanin biosynthesis from *Arabidopsis thaliana*, *Chrysanthemum morifolium*, and *Paeonia suffrutiosa* were aligned against MoMYB and MobHLH using BLAST (v2.14.0) (E-value < 1 × 10^−5^) implemented in Tbtools-II software [39]. Only sequences significantly similar to functionally validated anthocyanin-associated R2R3-MYB and bHLH proteins from these reference species were retained for further analysis. Subsequently, the conserved structural domains of the R2R3-MYB and bHLH members screened previously via the BLAST in TBtools-II were identified using the NCBI CDD tool. Multiple sequence alignments and visualizations of conserved sequences were performed using the DNAMAN 9.0. Based on the results of sequence alignment, a phylogenetic tree was constructed with MEGA 12.0 software by the neighbor-joining approach using 1000 bootstrap replicates. Homologous proteins from *Actinidia chinensis*, *A. thaliana*, *Camellia japonica*, *C. morifolium*, *Gentiana trifloral*, *Malus domestica*, *Oryza sativa*, *P. suffrutiosa*, *Rosa hybrid cultivar*, and *Vitis vinifera* were adopted for multiple sequence alignments and phylogenetic analysis.

### 2.6. Correlation Analysis Between TFs and Target Genes

Mantel matrix correlation analysis was performed to evaluate the overall transcriptional association between TFs related to anthocyanin biosynthesis and 33 anthocyanin biosynthetic structural genes. To calculate the global Mantel correlation coefficient and permutation *p*-value, Euclidean distance and 999 permutation tests were applied. Pairwise Mantel correlation coefficients and significance values between individual TFs and target genes are summarized in Table S1. A Mantel correlation butterfly heatmap was generated to visualize pairwise correlation patterns.

### 2.7. Quantitative Real-Time PCR (qRT-PCR)

To verify the reliability of DESeq, five key TFs and ten structural genes associated with flavonoid and anthocyanin synthesis were selected for qRT-PCR. The Megan RNA Extraction Kit (Guangzhou Magen Biotechnology Co., Ltd., Guangzhou, China) was utilized for isolating total RNA. The PrimeScript™ RT Reagent Kit with gDNA Eraser (Takara, Dalian, China) was employed for synthesizing the first-strand cDNA. The reaction product was subjected to ten-fold dilution with sterile water. The gene *Actin7* served as the endogenous reference for normalizing transcript levels [40]. The design of gene-specific primers was completed using the NCBI Primer-BLAST online approach (https://www.ncbi.nlm.nih.gov/tools/primer-blast/, accessed on 30 December 2022). More details of sequences are presented in Table S2. qRT-PCR amplification was conducted on LightCycler^®^ 480 II Real-Time System (LightCycler^®^ 480 II cycler, Roche, Carlsbad, CA, USA) using TB Green^®^ Premix Ex Taq™ II FAST qPCR (Takara, Dalian, China). qRT-PCR reactions were carried out in a 10 µL reaction volume, which consisted of SYBR Green Mix (5 µL), cDNA (4 µL), and respective primers (0.5 µL). The qRT-PCR amplification conditions were as follows: 30 s of initial denaturation at 95 °C, 5 s of denaturation at 95 °C, and 30 s of extension at 60 °C. When each experiment was completed, a melt-curve analysis was conducted with parameters set to 5 s at 95 °C and then 1 min at 60 °C. For every gene, three technical and biological replicates were prepared, and the 2^−ΔΔCT^ approach was employed for calculating relative gene expression levels [41].

## 3. Results

### 3.1. Identification and Quality Analysis of Anthocyanins

**Figure 2:**
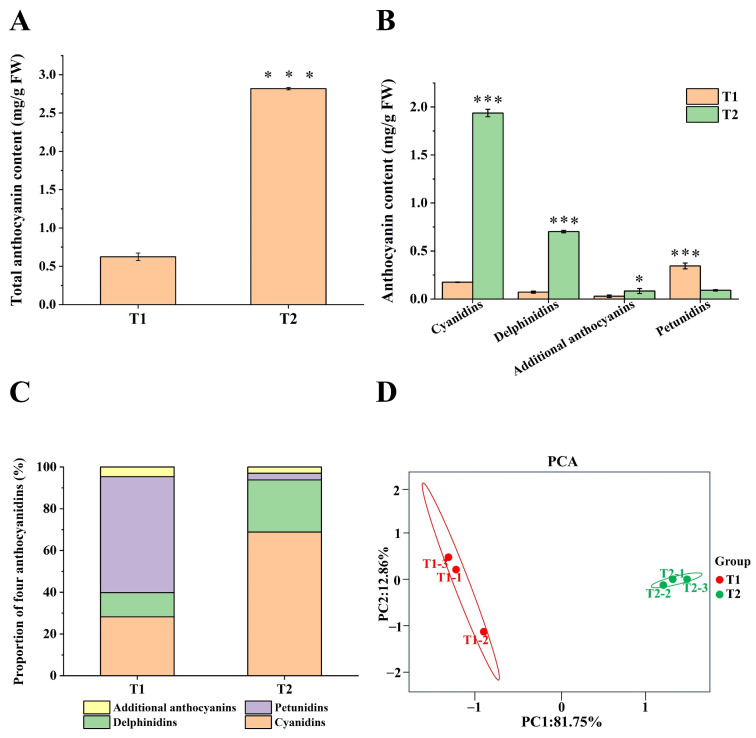
Identification and classification chart of anthocyanins. T1 and T2 represent light pink flowers and deep pink flowers, respectively. *, *p*-value < 0.05;- ***, *p*-value < 0.001. (**A**) The total anthocyanin content in T1 and T2. The *x*-axis and *y*-axis represent sample groups and total anthocyanin content, respectively. (**B**) The contents of four anthocyanin subclasses in T1 and T2. The *x*-axis and *y*-axis are anthocyanin types and their corresponding contents, respectively, while orange and green colors correspond to T1 and T2 groups. (**C**) The relative proportions of anthocyanin subclasses in T1 and T2. The *x*-axis and *y*-axis represent sample groups and the proportions of four anthocyanins, respectively, with orange, green, purple, and yellow colors corresponding to cyanidins, delphinidins, petunidins, and additional anthocyanins, respectively. (**D**) The PCA scatter plot. PC1 and PC2 stand for the first and second principal components, respectively, and the proportions indicate their corresponding variance explained. Red dots and green dots represent T1 and T2, respectively.

To explore how anthocyanins affected flower color change, anthocyanins in flower petals of *M. odora* with two colors were examined by high-performance liquid chromatography (HPLC) coupled with tandem mass spectrometry (MS/MS). The base peak chromatogram (BPC) and total ion chromatogram (TIC) of the two groups displayed obvious visual discrepancies across the full retention time range (Figures S1 and S2). A total of 117 flavonoids were annotated against mass spectrometry and public metabolite databases (Table S3), among which 18 were successfully identified. These anthocyanins were classified into four subclasses (Table S4): cyanidins (four compounds), delphinidins (five compounds), petunidins (two compounds), and additional anthocyanidins (seven compounds). The total anthocyanin content in deep pink flowers was significantly higher than that in light pink flowers, revealing the increasing total anthocyanin content within petals with deepening of color (Figure 2A). The contents of four anthocyanin subclasses were further quantified in two groups (Figure 2B). Consistent with total anthocyanin variation, cyanidins, delphinidins, and additional anthocyanidins exhibited higher average abundance in deep pink flowers than in light pink flowers. In order to compare the relative compositional difference in anthocyanin subclasses between the two groups, the proportional distributions of cyanidins, delphinidins, petunidins and additional anthocyanins in light pink and deep pink flowers were visualized side by side (Figure 2C). Distinct differences in relative anthocyanin composition could be observed between light and deep pink petals, indicating that the composition and relative proportions of anthocyanin are closely associated with petal color formation. Furthermore, samples from the two groups were subjected to PCA for assessing the differences in metabolites. The first (PC1) and second (PC2) principal components explained 81.75% and 12.86% of variation, respectively (Figure 2D). The OPLS-DA score plot illustrated the discrimination between the light pink and deep pink flower groups (Figure S3), with R2X = 0.938, R2Y = 1, and Q2 = 0.999, indicating that the model is reliable and stable for comparing metabolite accumulation between flowers of two colors. Overall, these results underscore significant variations in the accumulation of anthocyanins between the two groups.

### 3.2. Analysis of Differentially Accumulated Anthocyanins

**Figure 3:**
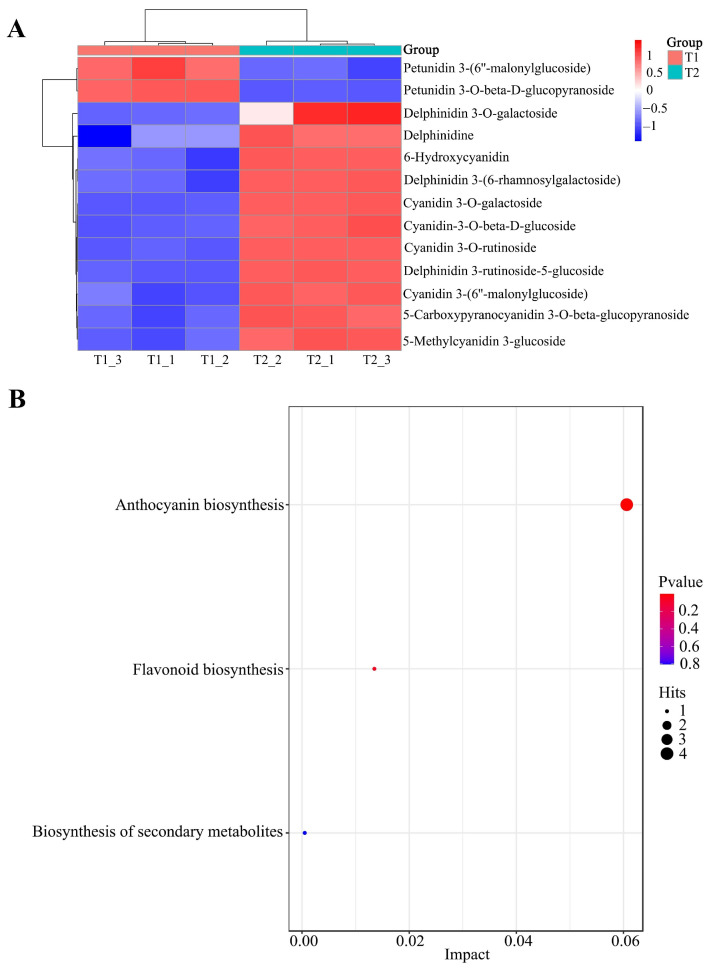
Differential anthocyanin analysis. T1 and T2 represent light pink flowers and deep pink flowers, respectively. (**A**) A hierarchical clustering heatmap of differential anthocyanins. In the heatmap, different colors represent the accumulation levels of each anthocyanin, where red to blue indicate high to low levels, respectively. (**B**) KEGG rich factor diagram of differential anthocyanins. Each dot stands for one metabolic pathway, where the *x*-axis indicates the impact values related to diverse metabolic pathways, whereas the *y*-axis indicates the enriched pathways. Dots size represents the number of metabolic molecules associated with this pathway. Color is associated with *p*-value, with a darker red color suggesting a lower *p*-value and a darker blue color indicating a greater *p*-value.

According to the screening standards of differential anthocyanin (*p*-value < 0.05, VIP value > 1, and FC ≥ 1.5), altogether 13 significantly different anthocyanins were identified between light pink and deep pink flowers. Among them, two were upregulated and the others were downregulated in light pink flowers (Table S5). The 13 anthocyanins were standardized for their metabolite contents, and then a cluster heatmap was drawn. The hierarchical clustering heatmap of differential anthocyanins showed that petunidin 3-(6″-malonylglucoside) and petunidin 3-O-beta-D-glucopyranoside exhibited significant expression within light pink flowers. Meanwhile, delphinidin 3-O-galactoside, delphinidin, delphinidin 3-(6-rhamnosylgalactoside), 6-hydroxycyanidin, cyanidin 3-(6″-malonylglucoside), cyanidin 3-O-galactoside, cyanidin-3-O-beta-D-glucoside, cyanidin 3-O-rutinoside, delphinidin 3-rutinoside-5-glucoside, 5-carboxypyranocyanidin 3-O-beta-glucopyranoside, and 5-methylcyanidin 3-glucoside displayed upward trends in deep pink flowers (Figure 3A).

As suggested by KEGG analysis, differential anthocyanins between the T1 and T2 groups were most closely related to the anthocyanin biosynthesis pathway (ko00942), followed by the flavonoid biosynthesis (ko00941) and secondary metabolite biosynthesis pathway (ko01110) (Figure 3B). This result suggested that the differential metabolites related to the anthocyanin biosynthesis pathway may have an important effect on color variations in both groups of *M. odora* flowers.

### 3.3. Transcriptome Sequencing and Analysis of DEGs

To detect genes related to anthocyanin accumulation within *M. odora* flowers, RNA-seq analysis was performed. Following quality control and raw data filtering, both clean read and clean data percentages reached above 93.29% (Table S6), and the GC content was at least 42.37% (Table S7). The functional annotation results showed the annotations of 33,663, 18,526, 12,623, 16,454, 34,566, and 22,880 unigenes by using NR, GO, KEGG, Pfam, eggNOG, and Swissprot databases, respectively (Table S8). From these results, the RNA-seq data were of high quality.

**Figure 4:**
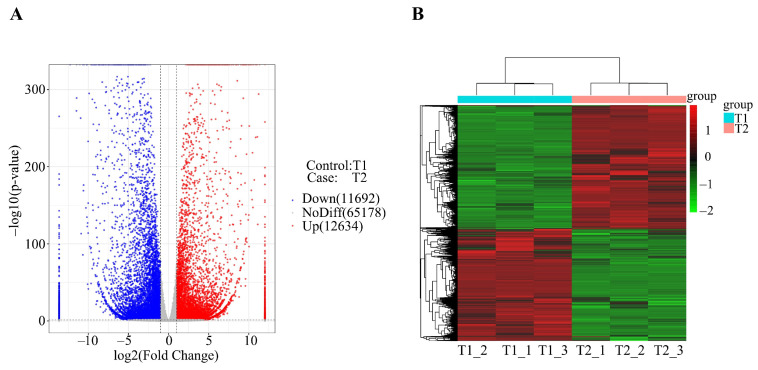
Analysis of DEGs. T1 and T2 represent light pink flowers and deep pink flowers, respectively. (**A**) Volcanic diagram of DEGs. Red, blue, and gray dots stand for upregulated, downregulated, and non-DEGs, respectively. (**B**) Cluster analysis of DEGs. The color scale indicates the normalized metabolite contents using Z-score. The horizontal axis represents genes, and each column represents a sample. Red color stands for high-expression genes and green color indicates low-expression genes.

Based upon the screening thresholds of |Log2FC| ≥ 1 and *p*-value < 0.05, 24,326 DEGs were identified in the *M. odora* flowers of two colors, including 12,634 upregulated and 11,692 downregulated genes (Figure 4A and Table S9). A heatmap illustrating DEG expression profiles among diverse samples was drawn. These samples were subjected to hierarchical clustering, which demonstrated that they were highly reproducible within groups, whereas DEG clustering revealed that gene modules were differentially expressed between groups (Figure 4B).

GO functional annotation results of DEGs were classified in molecular function (MF), biological process (BP), and cellular component (CC) categories. Of them, the top 10 significantly enriched GO terms or those showing lowest *p*-value are presented in Figure S4. Notably, enriched MFs were “UDP-glycosyltransferase activity” and “UDP-glucosyltransferase activity”, both of which are closely linked to the anthocyanin synthesis pathway. According to KEGG results, DEGs were predominantly abundant within various metabolic pathways (Figure S5), including starch and sucrose metabolism (ko00500), anthocyanin biosynthesis (ko00942), phenylpropanoid biosynthesis (ko00940), and flavonoid biosynthesis (ko00941). Among them, the starch and sucrose metabolism pathway is associated with plant biological metabolism and has a key effect on color formation. Phenylpropanoids serve as precursors for flavonoid biosynthesis and are essential for the synthesis of downstream compounds. The flavonoid biosynthesis pathway provides precursor metabolites to synthesize anthocyanins, while DEGs within this pathway play a key regulatory role, representing critical steps in anthocyanin synthesis.

### 3.4. Integrated Metabolomic and Transcriptomic Analyses

**Figure 5:**
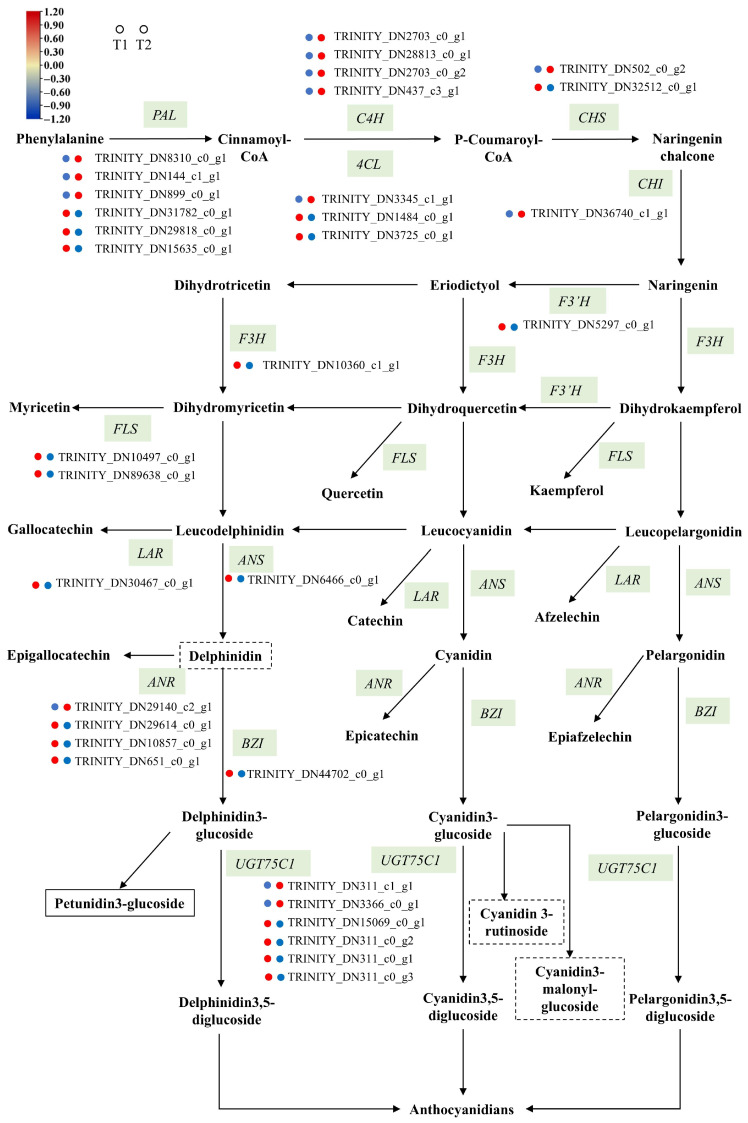
Integrated metabolomic and transcriptomics analyses on anthocyanidins. Pathway and structural genes associated with the biosynthesis pathways of phenylalanine, flavonoid, and anthocyanidin in *M. odora*, reconstructed on the basis of KEGG pathways enriched by DEGs. Green labels indicate catalytic enzymes related to the biosynthesis pathways of phenylalanine, flavonoid, and anthocyanidin. The color gradient bar at the top left represents Log2(FPKM) between light pink petals (T1) and deep pink petals (T2). Red and blue dots stand for genes with upregulation and downregulation, respectively. Solid and dotted black rectangular boxes represent upregulated and downregulated anthocyanin metabolites in T1, respectively. Enzymes involved in this pathway are as follows: *PAL*, phenylalanine ammonia-lyase; *C4H*, trans-cinnamate 4-monooxygenase; *4CL*, 4-coumarate-CoA ligase; *CHS*, chalcone synthase; *CHI*, chalcone isomerase; *F3H*, naringenin 3-dioxygenase; *F3′H*, flavanone 3′-hydroxylase; *ANS*, anthocyanidin synthase; *LAR*, leucoanthocyanidin reductase; *ANR*, anthocyanidin reductase; *FLS*, flavonol synthase; *BZI*, anthocyanidin 3-O-glucosyltransferase; *UGT75C1*, anthocyanidin 3-O-glucoside 5-O-glucosyltransferase.

Transcriptomic and metabolomic analyses were integrated for elucidating the anthocyanin biosynthesis pathway within the *M. odora* flowers (Figure 5). A total of four DAMs and thirty-three DEGs were identified as associated with the regulation of flower color. Starting from phenylalanine, dihydrokaempferol is synthesized through the catalysis of early biosynthetic genes including *MoPAL*, *MoC4H*, *MoC4L*, *MoCHS*, *MoCHI*, and *MoF3H*. Subsequently, colorless dihydrokaempferol is converted into various colored anthocyanins through the catalysis of enzymes such as *MoANS*, *MoBZI*, and *MoUGT75C1*. The KEGG-based annotation results indicated that delphinidin, cyanidin3-malonyl-glucoside, and cyanidin 3-rutinoside were downregulated in light pink flowers, whereas petunidin3-glucoside was upregulated. Indeed, the expression levels of some key genes exhibited significant changes, including three *MoPALs*, four *MoC4Hs*, one *Mo4CL*, one *MoCHS*, one *MoCHI*, one *MoANR*, and two *MoUGT75C1s*, which displayed higher expression abundances in deep pink flowers than in light pink flowers. The expression levels of these genes were positively related to anthocyanin accumulation within deep pink petals, including delphinidin, cyanidin 3-malonyl-glucide, and cyanidin 3-rutinoside (Figure 5), suggesting that these genes may regulate the transition of petal colors from light pink to deep pink in *M. odora* flowers. Conversely, the expression levels of three *MoPALs*, two *Mo4CLs*, one *MoF3H*, one *MoF3′H*, one *MoANS*, one *MoBZI*, and four *MoUGT75C1s* were significantly downregulated in their respective key pathways, implying that silencing these genes is essential for the change of petal color to deep pink. Interestingly, two *MoFLSs* were upregulated in the pathway from dihydroflavonols to flavonols in light pink flowers. Similarly, one *MoLAR* and three *MoANRs* were also significantly upregulated in the pathways of reducing colorless anthocyanins and colored anthocyanins to catechins and epicatechins, respectively. This indicates that these three types of genes may inhibit anthocyanin accumulation within light pink flowers.

### 3.5. Identification and Phylogenetic Classification of R2R3-MYB and bHLH TFs Related to Anthocyanin Biosynthesis

TFs play a crucial role in regulating anthocyanin biosynthesis and accumulation by modulating structural gene expression. In this work, altogether 2618 TF genes were identified through examining DEGs obtained based on transcriptomic annotations. Most TFs were classified into the bHLH, MYB-related, ERF, NAC, C2H2, WRKY, MYB, bZIP, C3H, and FAR1 families. Given that anthocyanin synthesis within plants is predominantly modulated via the R2R3-MYB and bHLH families [26,27,42,43], subsequent analyses focused on these two families. By screening homologs, three *MYB* and two *bHLH* homologs were identified based on the DEG analysis. For MYB, the sequence alignment confirmed that all three MoMYB proteins contained the typical R2R3-MYB domain possessing a bHLH interaction motif ([D/E]L × 2[R/K] × 3L × 6L × 3R) within the R3 repeat (Figure S6A). Notably, two MoMYBs (TRINITY_DN94722_c0_g1 and TRINITY_DN17123_c0_g1) possessed an EAR repression motif (L × L × L) in their C-termini like CmMYB4, AtMYB3, AtMYB4, or MdMYB111 (Figure S6B). Based on their amino acid sequence divergence, the R2R3-MYB proteins from the phylogenetic tree could be classified into activators and repressors (Figure S7). Furthermore, phylogenetic analysis showed that MoMYB (TRINITY_DN18853_c1_g1) clustered with GtMYB3 in *G. triflora* (Figure S7), and both were grouped into the activator clade. Meanwhile, another two MoMYBs (TRINITY_DN17123_c0_g1 and TRINITY_DN94722_c0_g1) clustered within the repressor clade of the phylogenetic tree. Specifically, MoMYB (TRINITY_DN17123_c0_g1) clustered with AtMYB4 and CmMYB4, while MoMYB (TRINITY_DN94722_c0_g1) exhibited a close evolutionary relationship with MdMYB111. For bHLH, the sequence alignment contained both the N-terminal MYB interaction region (MIR) and the bHLH domain (Figure S8A). Moreover, phylogenetic analysis revealed that MobHLH (TRINITY_DN8534_c0_g1) was closely related to GtbHLH1, while MobHLH (TRINITY_DN9917_c0_g1) clustered with AtGL3, AtEGL3, PsbHLH3, and CjbHLH1 (Figure S8B). Consequently, the three MYB genes (TRINITY_DN18853_c1_g1, TRINITY_DN94722_c0_g1, TRINITY_DN17123_c0_g1) were formally named MoMYB3, MoMYB4, and MoMYB111, respectively. Similarly, the two bHLH genes (TRINITY_DN8534_c0_g1, TRINITY_DN9917_c0_g1) were correspondingly designated as MobHLH1 and MobHLH3. Overall, it is likely that MoMYB3, MoMYB4, MoMYB111, MobHLH1, and MobHLH3 are related to regulating anthocyanin biosynthesis inside *M. odora* flowers.

### 3.6. Correlation Analysis Between MYBs/bHLHs and Anthocyanin-Related Genes

**Figure 6:**
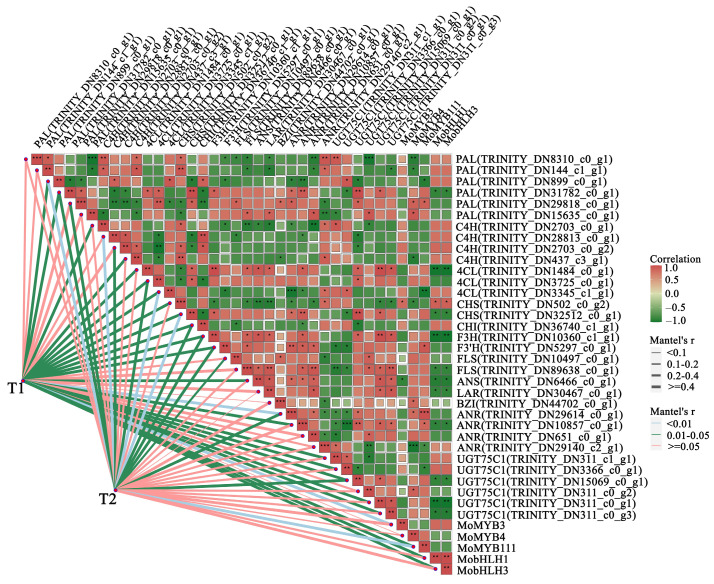
Mantel correlation butterfly heatmap between *MYB*/*bHLH* TFs and 33 DEGs. Red-green gradient was used in the central heatmap grid to reflect Pearson correlation coefficients (−1.0 to 1.0), with the color gradient from red to green representing the correlation from positive to negative. *, *p*-value < 0.05; **, *p*-value < 0.01; ***, *p*-value < 0.001. Line thickness corresponds to Mantel’s r, with thicker lines representing stronger correlations. Line color indicates Mantel’s *p*-value, and the color gradient from blue to pink corresponds to increasing *p*-values.

Mantel matrix correlation analysis was performed to evaluate the transcriptional linkage between five *MYB*/*bHLH* TFs and thirty-three DEGs associated with anthocyanin biosynthesis. The pairwise Mantel correlation heatmap indicated that core rate-limiting enzymes including three *PALs*, four *C4Hs*, one *4CL*, one *CHS*, one *CHI*, one *ANR*, and two *UGT75C1s* showed positive and extremely significant correlations with *MoMYB3*, *MobHLH1*, and *MobHLH3* (*p* < 0.05) (Figure 6), whereas these structural genes displayed negative correlations with *MoMYB4* and *MoMYB111*. These results suggest that anthocyanin biosynthesis is coordinately modulated by multiple TFs with divergent regulatory effects.

### 3.7. qRT-PCR of Transcriptomic Data

**Figure 7:**
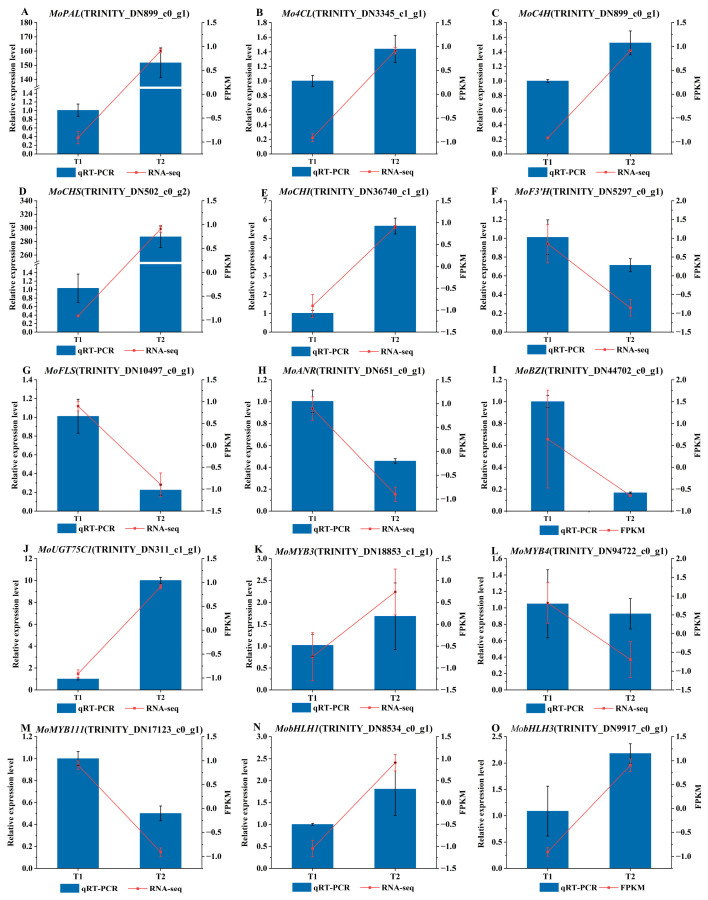
qRT-PCR validation of related expression levels of five key TFs and ten structural genes associated with flavonoid and anthocyanin synthesis in flowers of *M. odora*. T1 and T2 represent light pink flowers and deep pink flowers, respectively. The *x*-axis indicates two groups in this study, and the double *y*-axis stands for quantitative expression levels measured through qRT-PCR and the Z-score-normalized FPKM values obtained from RNA-seq, respectively.

To validate the accuracy of RNA-Seq findings, five TFs (*MoMYB3*, *MoMYB4*, *MoMYB111*, *MobHLH1*, and *MobHLH3*) and ten structural genes (*MoPAL*, *Mo4CL*, *MoC4H*, *MoCHS*, *MoCHI*, *MoF3′H*, *MoFLS*, *MoANR*, and *MoUGT75C1*) involved in flavonoid and anthocyanin synthesis were selected for qRT-PCR analysis. As indicated, *MoPAL*, *Mo4CL*, *MoC4H*, *MoCHS*, *MoCHI*, *MoUGT75C1*, *MoMYB3*, *MobHLH1*, and *MobHLH3* levels increased with the deepening of flower color, conforming to anthocyanin accumulation in deep pink flowers, whereas *MoF3′H*, *MoFLS*, *MoANR*, *MoBZI*, *MoMYB4*, and *MoMYB111* exhibited decreased expression levels (Figure 7). Notably, the expression levels of *MoPAL*, *MoCHS*, and *MoUGT75C1* remarkably increased in deep pink flowers compared with light pink flowers, with upregulation folds of 152, 287, and 10, respectively. The expression trends of these genes were consistent with sequencing findings, confirming that RNA-seq data are reliable and accurate.

## 4. Discussion

### 4.1. Anthocyanin Content in Diverse Colored Flowers of M. odora

Flower color serves as a significant ornamental trait that enhances the horticultural tree value as well as a critical adaptive phenotype during natural evolution [44]. Recently, more and more attention has been paid to the mechanism underlying flower color, with plant pigment content identified as a crucial factor influencing color diversity [45,46]. In many plant species, anthocyanin concentrations and compositions directly affect the coloration of plant tissues, particularly flower petals [47]. Peonidin and cyanidin have been previously suggested to confer a purplish-red hue, while pelargonidin is responsible for a brick-red color. Delphinidin, petunidin, and malvidin display a spectrum of colors from blue to purple [10,11]. For example, in the petals of different *Orychophragmus violaceus* cultivars, the highest contents of peonidin-3-glucoside and delphinidin 3-(6″-malonyl-glucoside) could be detected in purple flowers, followed by light purple and white varieties [48]. Peonidin 3-O-glucoside, cyanidin 3,5-O-diglucoside, and cyanidin 3-O-glucoside were identified within the pink petals of *Prunus mume*, whereas no compounds associated with the above secondary metabolites could be found in white petals [49]. Likewise, in the pink flowers of *M. maudiae*, a species belonging to the same genus as *M. odora*, cyanidin O-syringic acid, cyanidin 3,5-O-diglucoside, and cyanidin 3-O-glucoside were highly expressed [28]. In addition, it has also been found in *Camellia sinensis* that cyanidin and its glycosidic derivatives displayed particularly high concentrations in pink flowers [50]. In our study, metabolomic analysis identified 18 anthocyanins, of which 13 were obviously differentially expressed in the flowers of *M. odora* with different colors. Differential analysis revealed that deep pink petals exhibited markedly higher concentrations of anthocyanins than light pink petals, including four cyanidins, four delphinidins, and three additional anthocyanins. The results indicate that the difference in petal coloration between the two groups may be primarily attributed to variations in anthocyanin pigment content. Notably, compared with the four delphinidins and three additional anthocyanins, the four cyanidins accumulated to a greater extent in deep pink flowers, demonstrating that the four cyanidins may also be regarded as the key anthocyanins responsible for the deep pink color in *M. odora* flowers. In addition to cyanidins, delphinidins have also been reported to contribute to red coloration. Specifically, a decrease in delphinidin levels has been shown to inhibit red color formation [51,52]. Furthermore, delphinidin 3-O-glucoside was identified as a main anthocyanin within red flowers of *Catharanthus roseus* [53]. These findings suggested that the four delphinidins may also promote the deeper pink coloration observed in *M. odora* flowers. Furthermore, the three additional anthocyanins, namely 6-hydroxycyanidin, 5-carboxypyranocyanidin 3-O-β-glucopyranoside, and 5-methylcyanidin 3-glucoside, were detected in *M. odora* flowers. Studies have demonstrated that both 6-hydroxycyanidin and 5-carboxypyranocyanidin derivatives can promote red/orange-red coloration [54,55]. However, the pigment regulatory function of 5-methylcyanidin 3-glucoside remains unclear. This evidence taken together, we propose that the presence of the four cyanidins and four delphinidins, along with 5-carboxypyranocyanidin 3-O-β-glucopyranoside and 6-hydroxycyanidin, collectively contributes to the deep pink coloration in *M. odora* flowers.

### 4.2. Key Genes Affecting the Formation of Light Pink and Deep Pink Colors in the Anthocyanin Pathway

DEGs, particularly structural genes, play a crucial role in the production of various anthocyanin compounds. The biosynthetic pathway for anthocyanin originates from the phenylpropanoid pathway, which is initiated by enzyme-encoding genes such as *PAL*, *C4H*, and *4CL* [5,56]. Notably, the expression levels of *CHS* and *CHI* genes have been strongly related to anthocyanin content [57,58]. According to the above results, three *MoPALs*, four *MoC4Hs*, one *Mo4CL*, one *MoCHS*, and one *MoCHI* were significantly upregulated in deep pink flowers compared with light pink flowers, providing sufficient resources for anthocyanin biosynthesis. The upregulation of *F3′H*, *F3H*, *ANS*, and *BZI* have been identified as contributing to flower pigmentation in most species [50,59,60,61,62]. However, our transcriptomic analysis results suggested that the expression levels of *MoF3′H*, *MoF3H*, *MoANS*, and *MoBZI* exhibited downregulated levels in deep pink flowers. *F3′H*, *ANS*, *BZI*, and some *F3H* were observed to be in red leaves of *Acer triflorum* relative to orange leaves [62]. Additionally, the lack of *ANS* activities can lead to anthocyanin accumulation in tobacco [63]. These results implied that these structural genes did not consistently function as positive regulators across different species. Notably, this counterintuitive phenomenon that the lower expression of anthocyanin-promoting structural genes coincides with stronger pigmentation may be explained by a plausible mechanism supported by previous multi-omics research on flower coloration. Massive accumulation of anthocyanin end-products could initiate the transcriptional negative feedback to broadly repress core biosynthetic genes, thereby limiting continuous and excessive pigment synthesis. A similar regulatory pattern was reported in *Brassica napus* petals [5]. Therefore, it can be speculated that the downregulation of these genes in *M. odora* is caused by negative feedback triggered by excessive anthocyanin accumulation. The *Arabidopsis UGT75C1* mutant lacks anthocyanin 5-O-glucosides [64]. Furthermore, high expression of *UGT75C1* contributed to pelargonidin-3-Oglucoside formation in the pink flowers of *Hibiscus mutabilis* [65]. Only two out of six *MoUGT75C1* genes were upregulated in the deep pink petals in our study. Given the expression pattern of *MoUGT75C1* between the flowers of two colors, the regulatory effect of *UGT75C1* on anthocyanin accumulation that affects flower colors in *M. odora* needs to be further explored.

*FLS* converts dihydroflavonols into colorless or pale yellow flavonols [66,67]. *LAR* catalyzes the formation of catechin from leucoanthocyanidin [68,69], whereas *ANR* catalyzes the transformation of anthocyanidin into epicatechin [70]. Structurally, proanthocyanins are polymerized from varying amounts of catechins and epicatechins [71]. Functionally, proanthocyanidins mainly contribute to pale white and light yellow coloration in plant tissues [72]. Meanwhile, activation of the *LAR/ANR* pathway competitively inhibits anthocyanin accumulation, thereby weakening the formation of red, purple, and blue pigmentation [73]. Xia et al. [74] reported increased expression levels of *FLS* and *LAR* within white flowers relative to purple flowers of *Rhododendron pulchrum*. In *M. maudiae*, the high expression levels of *ANR* inside white flowers were consistent with the increasing trend of catechin and epicatechin contents [28]. In our study, one *MoFLS*, one *MoLAR*, and three *MoANR* genes were upregulated in light pink flowers. Collectively, these results suggest that the three competing pathway genes jointly consume precursors that otherwise flow into anthocyanin synthesis, and their coordinated upregulation shapes the light pink phenotype of *M. odora* flowers. In addition, differential anthocyanin profiling revealed that several anthocyanins, such as 5-methylcyanidin 3-glucoside, petunidin 3-(6″-malonylglucoside), petunidin 3-O-beta-D-glucopyranoside, and cyanidin 3-(6″-malonylglucoside), were related to methylation or acylation modification. However, DEGs associated with methyltransferase and acyltransferase have not been identified in our study based on transcriptomic analysis. Further research is required to identify and validate these relative genes involved in anthocyanin modification of *M. odora*.

### 4.3. Analysis of TFs Associated with Anthocyanin Biosynthesis Pathway

In addition to the immediate functions of structural genes, TFs also exert important effects on the regulation of anthocyanin biosynthesis, including three main classes: R2R3-MYB TFs, bHLH TFs, and WD40-repeat proteins [43,75]. Of these, R2R3-MYB TFs have been determined to regulate anthocyanin biosynthesis [26,42,43]. On one hand, R2R3-MYB has been demonstrated to negatively regulate anthocyanin biosynthesis among various plants [27,76,77,78]. It has been well documented that the EAR motif confers repressive activity on anthocyanin biosynthesis, as evidenced in various R2R3-MYB members such as *AtMYB4* from *A. thaliana*, *CmMYB4* from *C. morifolium*, and *MdMYB111* from *M. domestica* [76,77,78]. From our results, MoMYB4 and MoMYB111 contained EAR motifs at their C-termini and had relatively higher homology with the AtMYB4, CmMYB4, and MdMYB111, respectively. Given their lower expression levels in deep pink flowers according to qRT-PCR verification, these *MoMYB* genes may also function as repressors of anthocyanin biosynthesis via their EAR motifs.

On the other hand, R2R3-MYB can also positively regulate anthocyanin biosynthesis by the MYB-bHLH dimer or MYB-bHLH-WD40 trimer (MBW) [42,43,75]. For example, it has been reported that *GtMYB3* can interact with *GtbHLH1* to form a transcriptional regulatory complex, which jointly activates the expression of genes associated with anthocyanin synthesis and positively regulates gentiodelphin within *G. triflora* petals [79]. Similar MYB-bHLH synergistic interactions, which affect flower color via anthocyanin regulation, have been documented in *Dendrobium* hybrids, rose, and *Freesia hybrida* [80,81,82]. Here, *MoMYB3* and *MobHLH1* showed high expression in deep pink petals as revealed by qRT-PCR verification and shared close phylogenetic relationships with GtMYB3 and GtbHLH1 from *G. triflora*, respectively. Additionally, MoMYB3 protein contained a signature motif that was predicted to interact with bHLH. Meanwhile, MobHLH1 protein also processed the interaction region with MYB. Therefore, it was speculated that *MoMYB3* and *MobHLH1* may form a similar functional complex and jointly regulate the anthocyanin synthesis in *M. odora*, further regulating the flower color. Moreover, *AtGL3* and *AtEGL3* each act as a key bHLH partner within the MBW complex in *A. thaliana*, redundantly activating anthocyanin biosynthetic genes like *F3′H*, *DFR*, and *LDOX* [83]. In our study, MobHLH3 shared the closest evolutionary relationship with the AtGL3/AtEGL3-type bHLH clade, and its expression trend was correlated positively with the anthocyanin content in deep pink flowers as confirmed by qRT-PCR validation, implying that *MobHLH3* may form an MBW complex with specific *MYB* and WD40 partners, thereby activating anthocyanin biosynthetic genes in *M. odora* flowers. Furthermore, correlation analysis revealed that four *C4Hs*, one *4CL*, one *CHS*, one *CHI*, one *ANR*, and two *UGT75C1s* were positively correlated with *MoMYB4*, *MobHLH1*, and *MobHLH3*, whereas they were negatively correlated with *MoMYB111* and *MoMYB3*. The results provide preliminary transcriptional evidence for the regulatory association between *MYB*/*bHLH* TFs and anthocyanin biosynthetic structural genes in *M. odora*. However, the interactions and regulatory mechanisms between *MYBs*/*bHLHs* and structural genes need to be further elucidated.

## 5. Conclusions

In the present work, metabolomic and transcriptomic analyses were integrated for elucidating differential regulation of anthocyanin biosynthesis in *M. odora* flowers with light pink and deep pink colors. A total of 13 anthocyanins with differential accumulation were identified. Among these, 11 anthocyanins, particularly cyanidins, were likely the key components that determined the deep pink color, while the reduced anthocyanin content contributed to the formation of light pink color. Furthermore, transcriptomic analysis showed that 33 structural genes were identified in the flavonoid and anthocyanin biosynthesis pathway. High expression levels of *MoPAL*, *MoC4H*, *Mo4CL*, *MoCHS*, *MoCHI*, and *MoUGT75C1* genes might play positive roles in the anthocyanin accumulation of deep pink flowers. Meanwhile, the upregulation of *MoFLS*, *MoLAR*, and *MoANR* was likely responsible for the formation of light pink color. Furthermore, *MoMYB3* and *MobHLH1* were predicted to positively regulate anthocyanin accumulation, while *MobHLH3* might act as a participant of the MBW complex in promoting pigmentation. The above results lay a theoretical basis for the molecular breeding of *M. odora* aimed at increasing anthocyanin contents and diverse petal colors.

## Data Availability

All data generated in this research are included within the article. The raw data of RNA-seq were deposited in the NCBI SRA database under accession number PRJNA1476995.

## Supplementary Materials

The following supporting information can be downloaded at: https://www.mdpi.com/article/10.3390/biology15141217/s1. Table S1: Mantel correlation coefficients and significance values between five TFs and structural genes. Table S2: Primer sequences used for quantitative real-time PCR. Table S3: Quantitative summary of anthocyanins for primary and secondary identification. Table S4: Identification and quantification of anthocyanins. Table S5: Screening results of differential anthocyanins. Table S6: Statistics of filtered data based on RNA-seq. Table S7: Statistics of sequence population based on RNA-seq. Table S8: Functional annotation results of unigenes. Table S9: DEGs between T1 and T2. Figure S1: Base peak chromatograms of T1 and T2. Figure S2: Total ion chromatograms of T1 and T2. Figure S3: OPLS-DA score plot. Figure S4: The top 10 GO terms based on DEGs. Figure S5: KEGG enrichment *p*-value barplot chart of T1 vs T2. Figure S6: Characterization analysis of MoMYB protein. Figure S7: Phylogenetic analysis of three MoMYBs and their homologs from other plants. Figure S8: Characterization analysis of MobHLH protein.
