# Supplementary figures and images for: Integrated Metabolome and Transcriptome Analysis Reveals the Effect of Anthocyanins on Flower Color Variation in Michelia odora
Source: Biology (Basel). 2026 Jul 22;15(14):1217. doi: 10.3390/biology15141217 (PMC13404592; doi:10.3390/biology15141217)

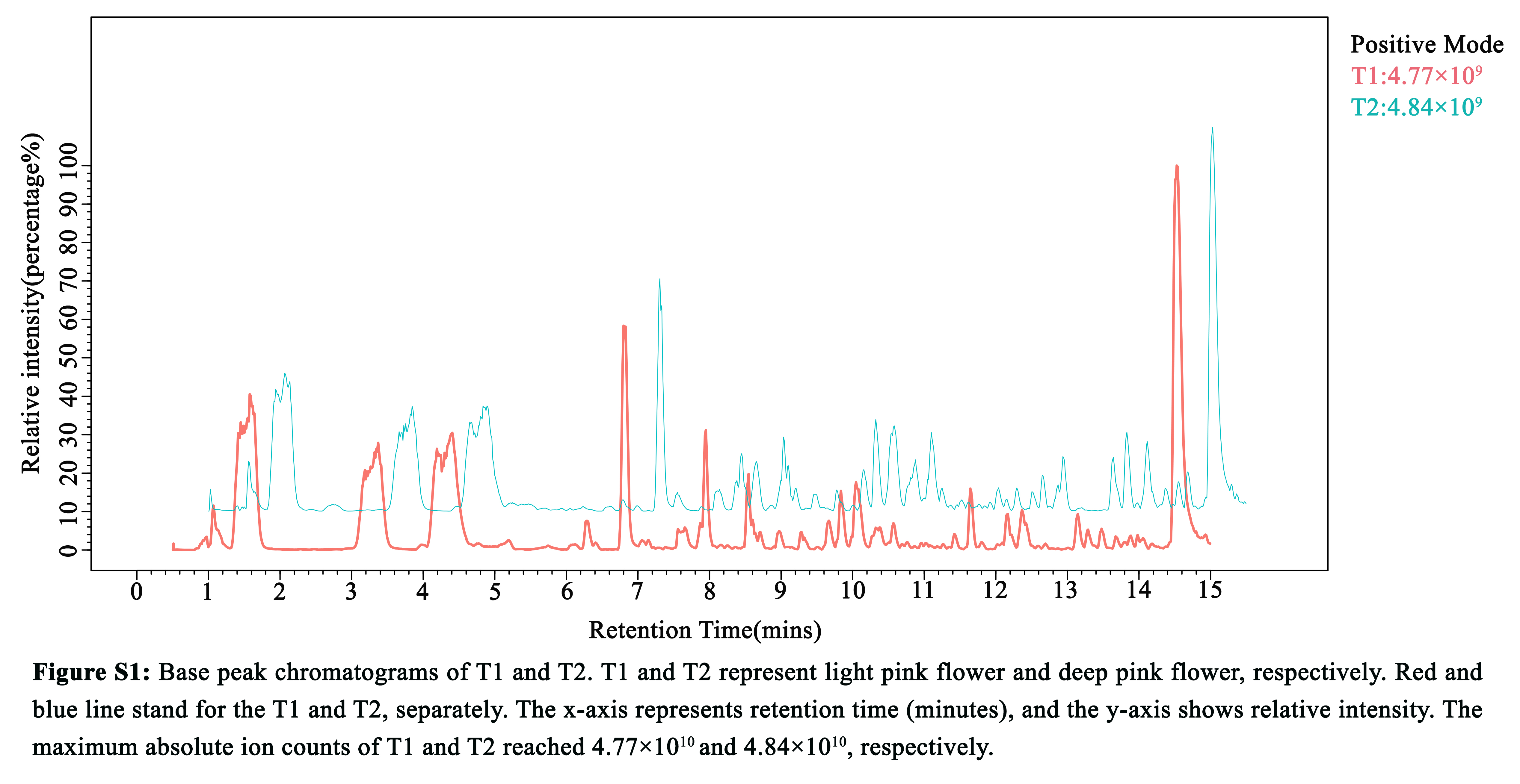

Supplement: Supplementary file 1 [file biology-15-01217-s001.zip › Figure S1.jpg]

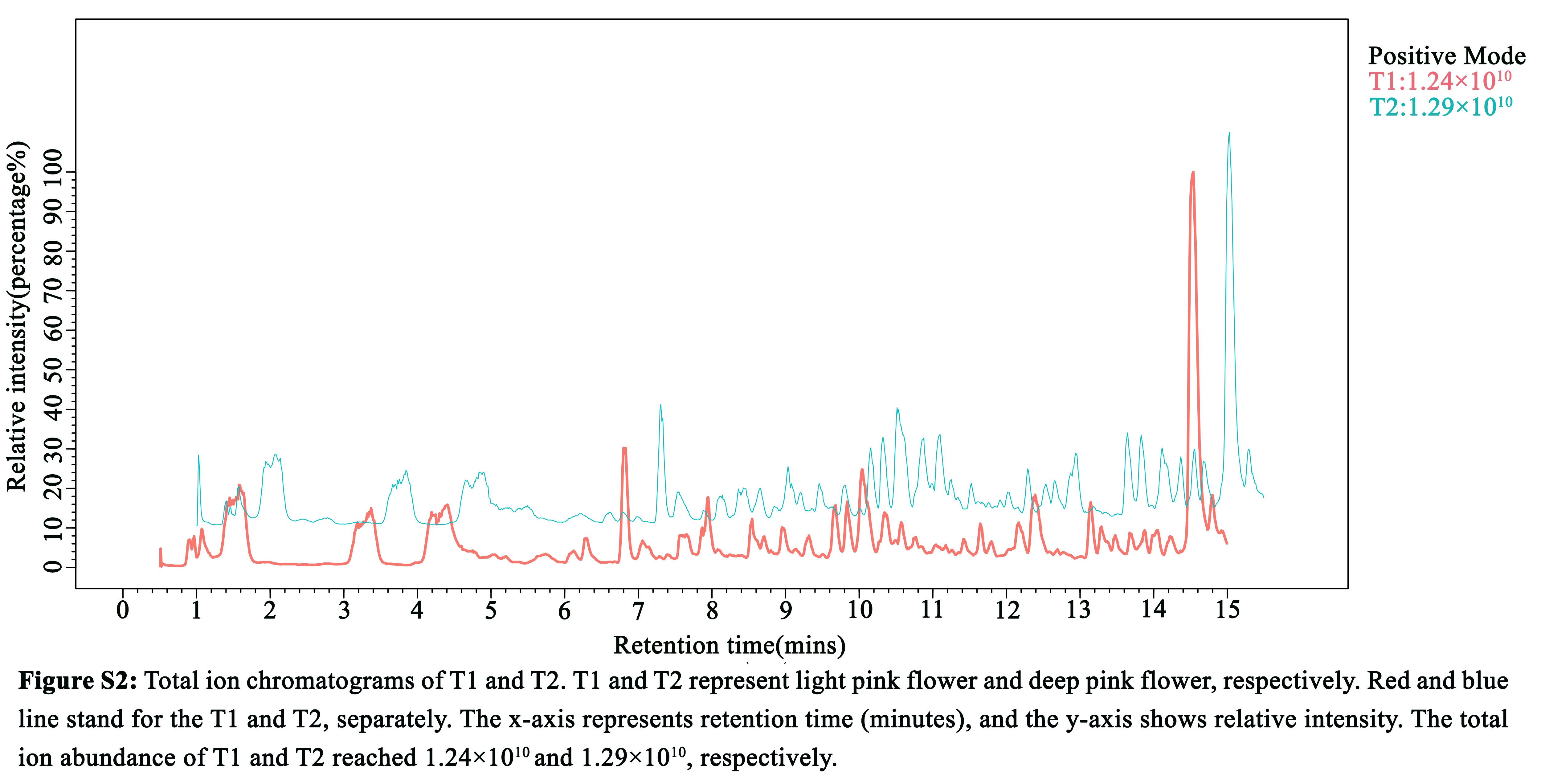

Supplement: Supplementary file 1 [file biology-15-01217-s001.zip › Figure S2.jpg]

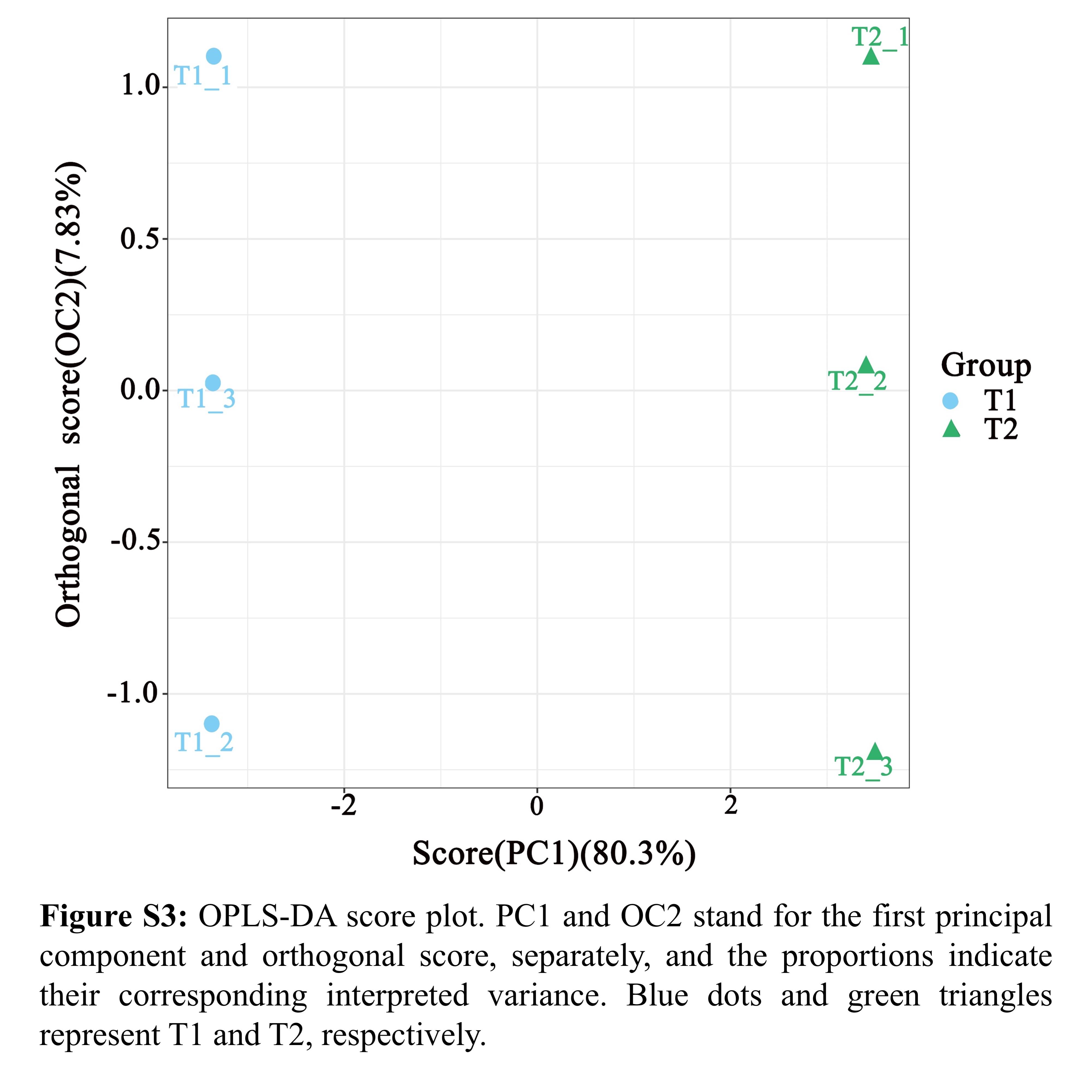

Supplement: Supplementary file 1 [file biology-15-01217-s001.zip › Figure S3.jpg]

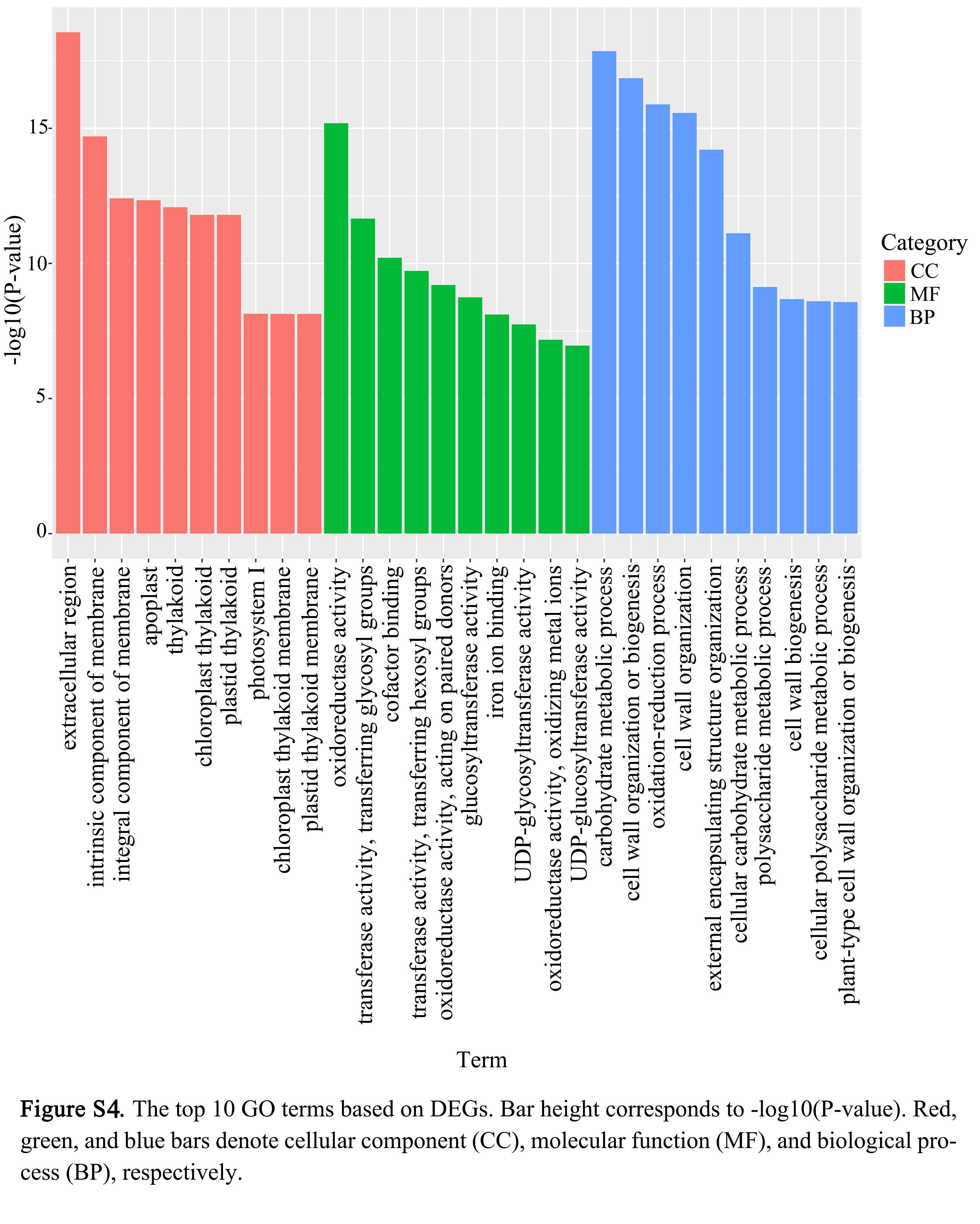

Supplement: Supplementary file 1 [file biology-15-01217-s001.zip › Figure S4.jpg]

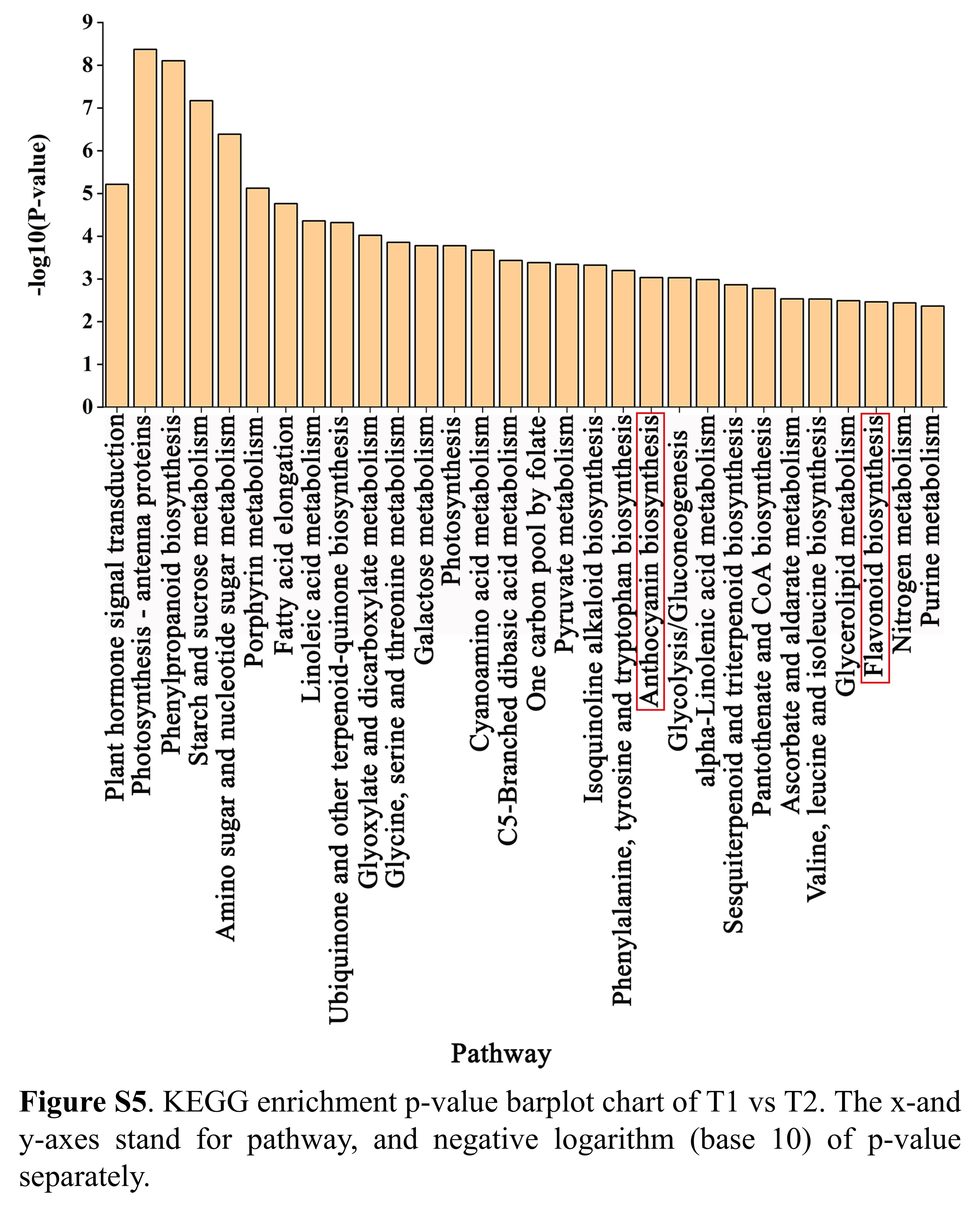

Supplement: Supplementary file 1 [file biology-15-01217-s001.zip › Figure S5.jpg]

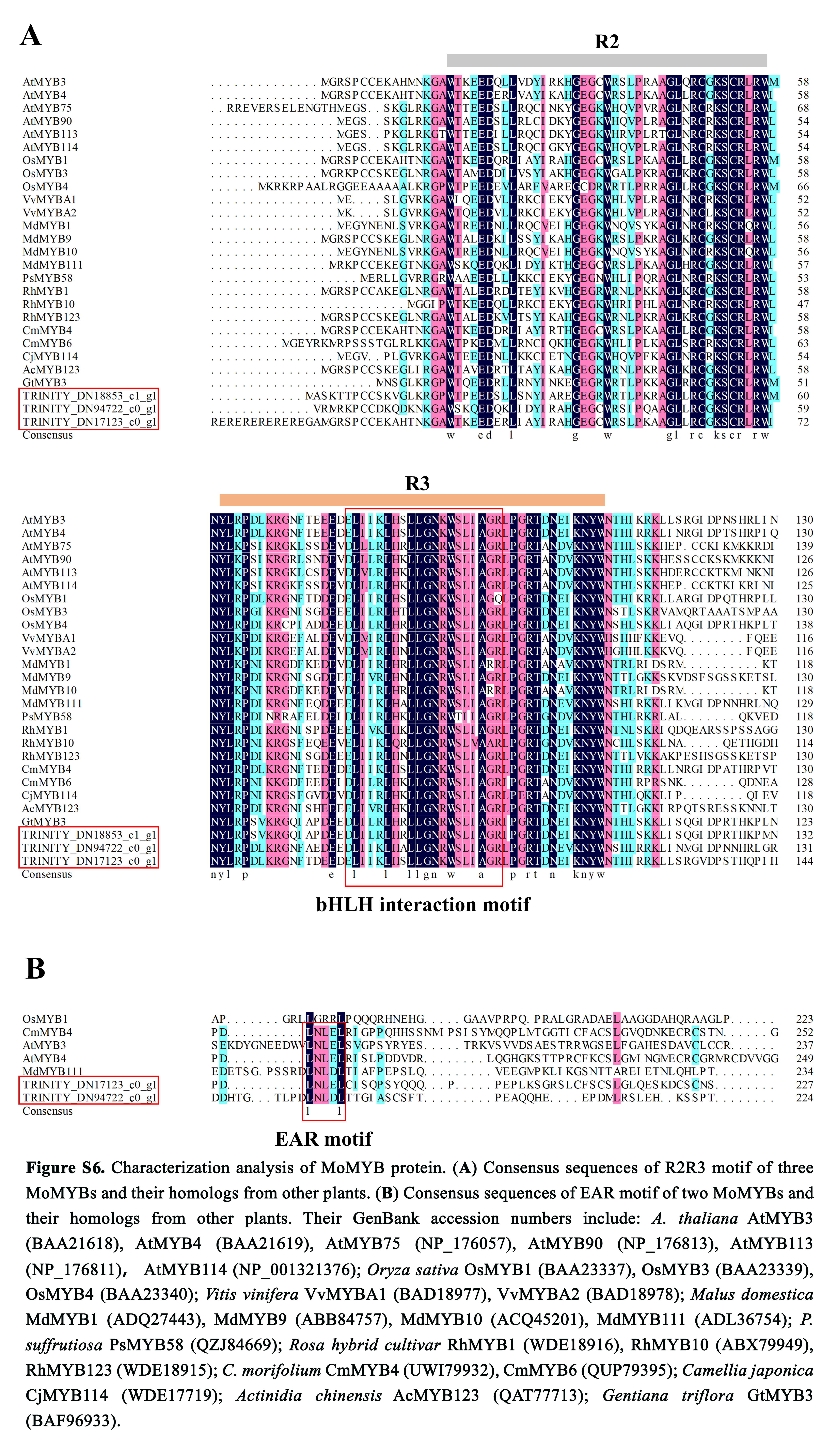

Supplement: Supplementary file 1 [file biology-15-01217-s001.zip › Figure S6.jpg]

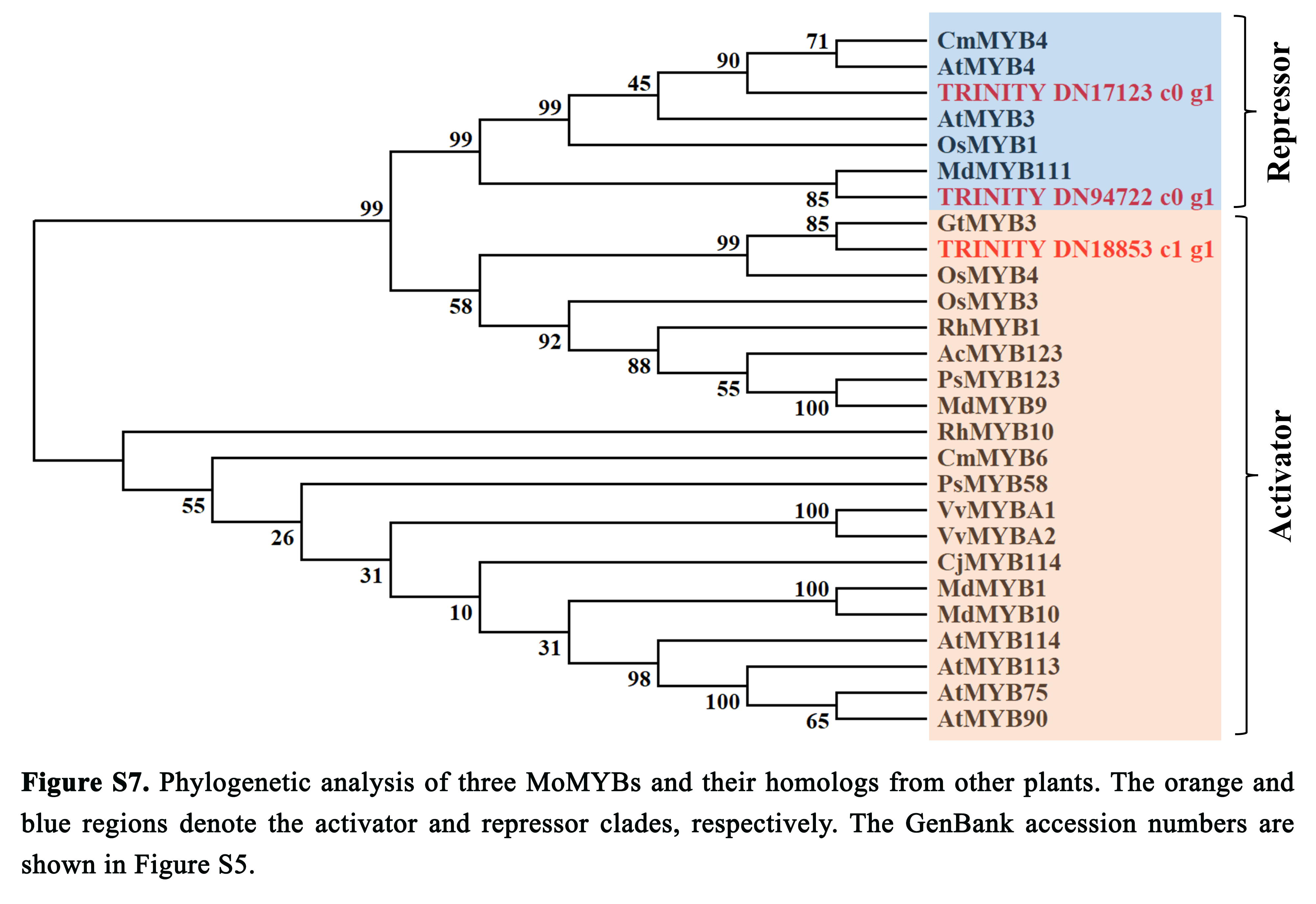

Supplement: Supplementary file 1 [file biology-15-01217-s001.zip › Figure S7.jpg]

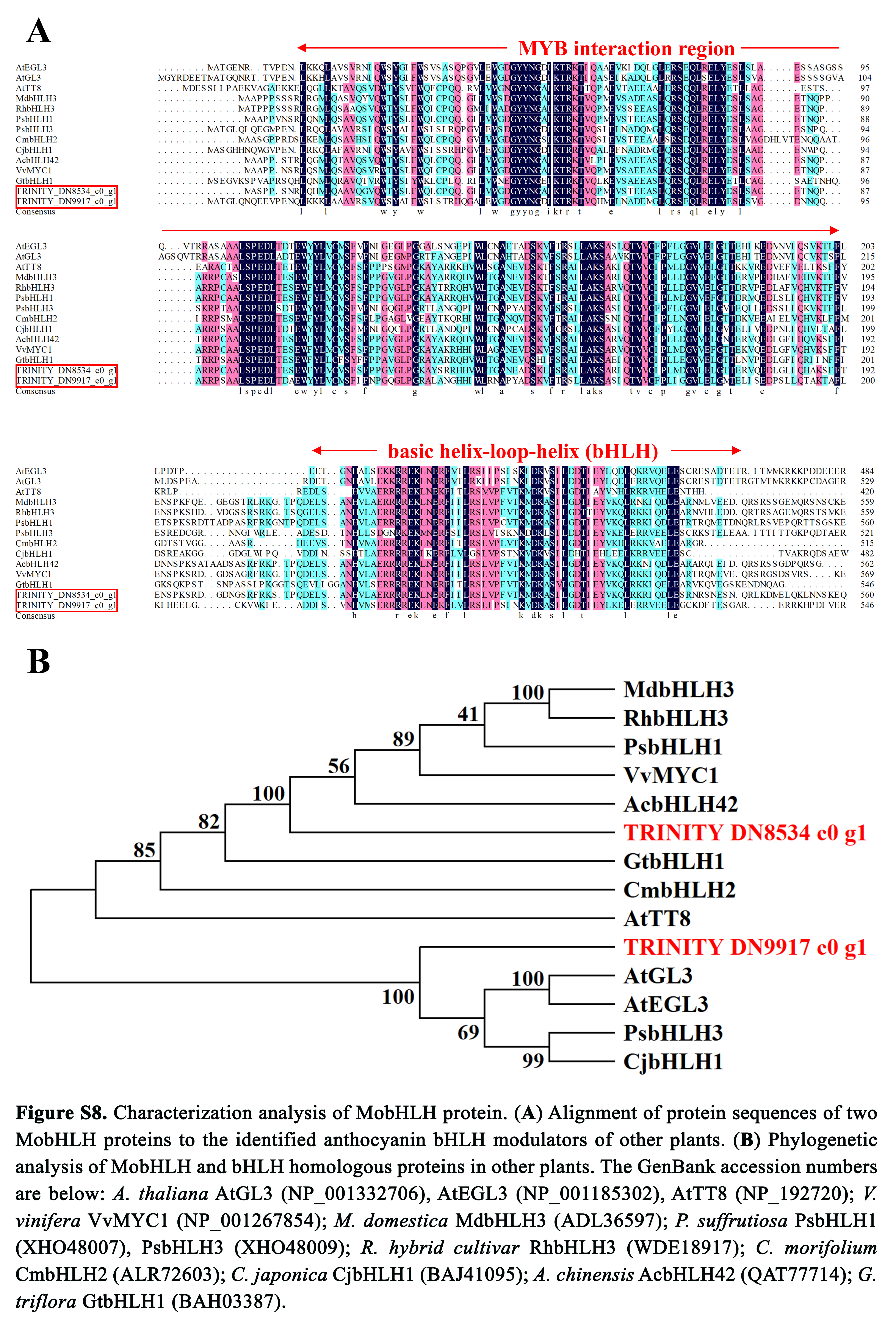

Supplement: Supplementary file 1 [file biology-15-01217-s001.zip › Figure S8.jpg]
